# Detection of Active BoNT/C and D by EndoPep-MS Using MALDI Biotyper Instrument and Comparison with the Mouse Test Bioassay

**DOI:** 10.3390/toxins13010010

**Published:** 2020-12-24

**Authors:** Ilenia Drigo, Elena Tonon, Simone Pascoletti, Fabrizio Anniballi, Suzanne R. Kalb, Luca Bano

**Affiliations:** 1 Special Bacteriology Laboratory, SCT2—Istituto Zooprofilattico Sperimentale delle Venezie, Vicolo Mazzini 4, 31020 Fontane di Villorba, Italy; etonon@izsvenezie.it (E.T.); simonepascoletti@gmail.com (S.P.); lbano@izsvenezie.it (L.B.); 2 National Reference Centre for Botulism, Microbiological Foodborne Hazard Unit., Department of Food Safety, Nutrition and Veterinary Public Health, Istituto Superiore di Sanità, viale Regina Elena 299, 00161 Rome, Italy; fabrizio.anniballi@iss.it; 3 Centers for Disease Control and Prevention, National Center for Environmental Health, Division of Laboratory Sciences, 4770 Buford Hwy NE, Atlanta, GA 30341, USA; ssk7@cdc.gov

**Keywords:** *Clostridium botulinum*, BoNT/C, BoNT/D, BoNT/CD, BoNT/DC, MALDI Biotyper

## Abstract

Botulinum neurotoxins (BoNTs) are among the most poisonous known biological substances, and therefore the availability of reliable, easy-to use tools for BoNT detection are important goals for food safety and human and animal health. The reference method for toxin detection and identification is the mouse bioassay (MBA). An EndoPep-MS method for BoNT differentiation has been developed based on mass spectrometry. We have validated and implemented the EndoPep-MS method on a Bruker MALDI Biotyper for the detection of BoNT/C and D serotypes. The method was extensively validated using experimentally and naturally contaminated samples comparing the results with those obtained with the MBA. Overall, the limit of detection (LoD) for both C and D toxins were less than or equal to two mouse lethal dose 50 (mLD_50_) per 500 µL for all tested matrices with the exception of feces spiked with BoNT/C which showed signals not-related to specific peptide fragments. Diagnostic sensitivity, specificity and positive predictive value were 100% (95% CI: 87.66–100%), 96.08% (95% CI: 86.54–99.52%), and 93.33% (95% CI: 78.25–98.20%), respectively, and accuracy was 97.47% (95% CI: 91.15–99.69%). In conclusion, the tests carried out showed that the EndoPep-MS method, initially developed using more powerful mass spectrometers, can be applied to the Bruker MALDI Biotyper instrument with excellent results including for detection of the proteolytic activity of BoNT/C, BoNT/D, BoNT/CD, and BoNT/DC toxins.

## 1. Introduction

Botulinum neurotoxins (BoNTs) are proteins produced by Gram-positive, rod-shaped, spore-forming, anaerobic bacteria belonging to *Clostridium* (*Clostrisium botulinum*, *Clostrisium butyricum*, *Clostridium baratii*, *Clostrisium argentinense,* and *Clostridium sporogenes*) [1]. In recent years, BoNT-like encoding genes have also been detected in the genome of non-*Clostridium* species, such as *Weissella oryzae* [2], *Enterococcus* spp. [3], and *Chryseobacterium piperi* [4]. The specific features of BoNT production by these microbes are unknown, as well as their possible interactions in ecological niches. There are seven distinct serotypes of BoNTs from A (BoNT/A) through G (BoNT/G) based on their antigenic properties, and many serotypes contain subtypes or variants [5,6]. In addition to these seven serotypes, a chimeric BoNT type H, also called FA or HA, and a putative novel type, called X, have been recovered in *Clostridium botulinum* (C. botulinum) previously classified as type B strain [7,8]. BoNT/A, B, E, and F mainly cause human disease, whereas BoNT/C and D have been shown to affect animals [2,6,9]. The neuroparalytic syndrome resulting from the systemic effects of BoNTs is potentially fatal so it is crucial to detect BoNTs as early as possible in a sample to prevent additional botulism cases and provide, when possible, the target treatment. The test most widely used to confirm the presence of BoNTs and identification them is the mouse bioassay (MBA) [10]. This lethality assay possesses high sensitivity, can measure toxin activity, and can detect toxin serotype by means of specific antitoxins. However, it does pose ethical issues because it requires the sacrifice of many animals, it is time consuming since four days are needed for confirmation of negative samples, and it requires a large volume of sample [11]. Furthermore, other substances in the sample, e.g., other toxins or irritating ingredients such as spices in foods, can occasionally lead to inconclusive results [12,13]. Many efforts have been made in recent years to develop alternative laboratory methods to the mouse lethality test and some are very promising. Most developed tests have proven to be less sensitive than the MBA and/or been unable to give information about toxin activity (e.g., ELISA tests). Recently, an EndoPep-MS method for BoNTs and serotype differentiation based on LC-ESI-MS/MS and MALDI-TOF MS, coupled with antibody purification and enrichment of toxins, was developed and has been successfully applied in various types of samples for detection of BoNT/A, B, C, D, E, F, and G [14,15,16]. The original method was developed using costly, high-resolution mass spectrometers that are rarely present in routine diagnostic laboratories, not only because of their high cost but, also, because they need personnel with high technical skills to use them. In 2017, Perry and coworkers [11] implemented the EndoPep-MS method for the detection of BoNT/A, B, E, and F toxins using the Bruker MALDI Biotyper. This is a lower performance instrument compared with other more costly ones, but is commonly found in microbiology laboratories, in both the human and veterinary fields, being routinely dedicated to bacterial identification. The rapid diffusion of MALDI-TOF MS technology in a growing number of diagnostic laboratories worldwide is due to its versatility, rapidity of analysis, lower of consumable costs, high-throughput, and easy handling [17]. Here we describe the application and validation of the EndoPep-MS method to detect of proteolytic activity of BoNT/C and D and their mosaic forms CD and DC in animal clinical specimens, using the Bruker MALDI Biotyper. We compared the test performance of EndoPep-MS with the MBA test, considered the “gold standard” for botulinum neurotoxin detection.

## 2. Results

### 2.1. Limit of Detection (LoD)

**Figure 1:**
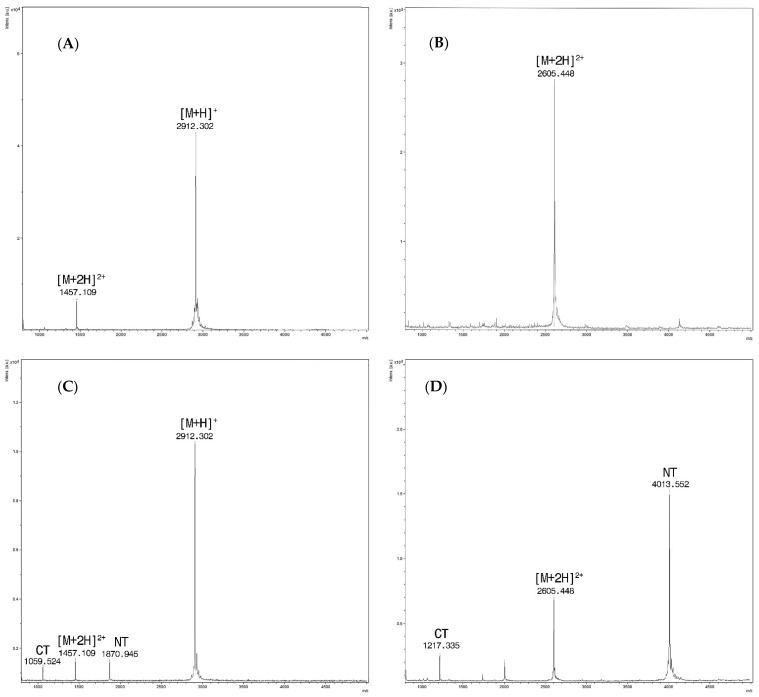
(**A**) Sera spiked only with PepC, at m/z 2912.3 the singly charged ion [M + H]^+^ and at m/z 1457.1 for the doubly charged one [M + 2H]^2+^ **;** (**B**) Sera spiked only with PepD/F. Only the doubly charged ion at m/z 2605.4 [M + 2H]^2+^ is detected within the range of analysis; (**C**) Spectra from the reaction of sera spiked with BoNT/C at a concentration of 16 mLD_50_/500 µL and Pep C (15 mM). Intact PepC at m/z 2912.3 [M + H] ^+^ with the doubly charged ion [M + 2H]^2+^at m/z 1457.1 and cleavage products at m/z 1870.9 (NT) and m/z 1059.5 (CT). (**D**) Spectra from the reaction of sera spiked with BoNT/D at a concentration of 16 mLD_50_/500 µL and Pep D/F (15 mM). Doubly charged PepD/F [M + 2H]^2+^ at m/z 2605.4 and cleavage products at m/z 4013.5 (NT) and m/z 1217.3 (CT), respectively.

**Figure 2:**
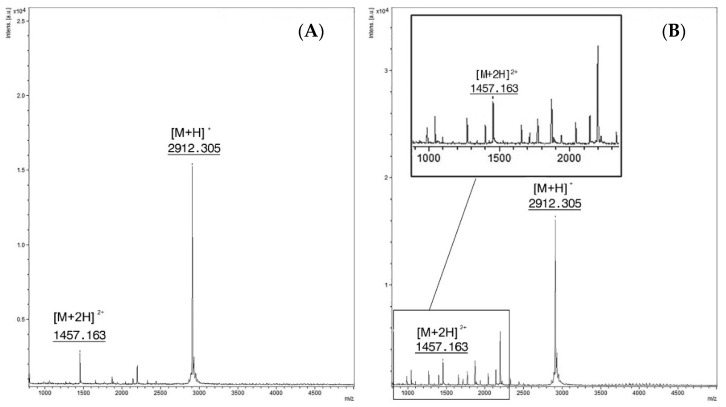
(**A**) Feces spiked with PepC. Singly charged ion at m/z 2912.3 [M + H]^+^ and the doubly charged one at m/z 1457.1 [M + 2H]^2+^ (**B**) Spectra from the reaction of feces spiked with BoNT/C at a concentration of 16 mLD_50_/500 µL and PepC (15 mM). Intact PepC at m/z 2912.3 [M + H]^+^ with the doubly charged ion [M + 2H]^2+^at *m/z* 1457.1, cleavage products are not clearly visible.

In order to determine the LoD of EndoPep-MS, two-fold serial dilutions of BoNT/C and D reference toxins were used to contaminate water, serum, broth culture from feces, and feces diluted to 1:1 in gelatin phosphate buffer (GPB). The contaminated specimens were then analyzed by the EndoPep-MS method and the results are described in Table 1. An example of mass spectra obtained from sera spiked with C and D toxins is reported in Figure 1. For BoNT/C, all the contaminated matrices gave an LoD lower or equal to 2 mLD_50_/500 µL, except for feces in GPB for which nonspecific peaks were observed, preventing the typical peptide typical peaks from being visualized (Figure 2). For BoNT/D, the LoDs for toxin spiked in water and sera were overall equal to or lower than 1 mLD_50_ /500 µL, but were equal to 2 mLD_50_/500 µL or lower for broth culture of feces. Feces showed an LoD of about 2 mLD_50_/500 µL without interference from fecal inhibitors.

### 2.2. Specificity

The method was able to detect BoNT activity only in broth culture of clostridia producing type C, C/D, D, and D/C toxins. No cross reactivity was observed with the other enrichment cultures of neurotoxin-producing types A, B, E, and F clostridia (Table 2).

### 2.3. Comparison of LoD between the EndoPep-MS Method and the MBA

To measure the EndoPep-MS method against the MBA, sera, broth culture, and feces diluted with GPB to 1:1 were spiked with BoNT/C and BoNT/D reference toxins at final concentrations of 4, 2, 1, 0.5, and 0.25 mLD_50_/500 µL. Spiked and non-spiked samples were tested cat the same time with both methods and the results were compared (Table 3).

The EndoPep-MS method developed to detect BoNT/C in serum was able to identify the target at a 2-fold higher concentration than the MBA (1 mLD50/500 µL vs. 0.5 mLD50/500 µL). Conversely, the LoD of our EndoPep-MS method applied to broth cultures was lower than the MBA. This latter result guests that the MBA was slightly less sensitive than EndoPep-MS (2 mLD50/500 µL vs 4mLD50/500 µL). In the feces spiked with BoNT/C, the presence of nonspecific peaks prevented comparison between the two tests as observed in sensitivity tests. The LoD of the MBA was in this case 4 mLD50/500 µL. The LoD of BoNT/D in serum and feces were 2-fold lower for EndoPep-MS than for the MBA (1 mLD50 /500 µL vs. 2 mLD50/500 µL for serum and 2 mLD50/500 µL vs. 4 mLD50/500 µL for feces, respectively) whereas a higher LoD was observed for EndoPep-MS than for the MBA (2 mLD50 /500 µL vs. 4 mLD50/500 µL) in the case of broth culture.

### 2.4. Diagnostic Sensitivity and Specificity

Diagnostic sensitivity and specificity were evaluated by testing naturally contaminated samples collected during routine laboratory activities by Istituto Zooprofilattico Sperimentale delle Venezie and the National Reference Centre for Botulism (CNRB) of the Italian Istituto Superiore di Sanità. The “gold standard” used for diagnosis was the MBA and the results for the enrichment cultures were confirmed by multiplex real-time PCR. In total, 79 samples were analyzed and the results are reported in Table 4. The two sera samples 2659/4/10 and 6841/3/13 obtained from subjects of two confirmed animal botulism outbreaks, tested negative with the MBA but proved to be weakly positive with EndoPep-MS in both replicates.

The sensitivity of EndoPep-MS was 100% (95% CI: 87.66–100%) and the specificity 96.08% (95% CI: 86.54–99.52%), with a positive predictive value of 93.33% (95% CI: 78.25–98.20%), a negative predictive value of 100%, and accuracy of 97.47% (95% CI: 91.15–99.69%).

## 3. Discussion

Mass spectrometry has become an important analytical tool for many applications in microbiology, especially for rapid, precise identification of pathogens in clinical microbiology laboratories. Numerous studies have demonstrated that MALDI-TOF MS surpasses conventional diagnostic methods in terms of cost, speed, and accuracy in the identification of microbial species [19]. These characteristics have led to the rapid spread of lower-performance mass spectrometers than the ones used in chemistry laboratories, which show great potential for use in other activities, as, strain typing, antimicrobial susceptibility testing and bacterial toxin detection, including BoNTs [20].

The current “gold standard” for BoNTs detection is the MBA but the use of laboratory animals poses a non-negligible ethical issues and should be avoided when alternative methods are available. Researchers from CDC developed an EndoPep-MS method for the identification and evaluation of BoNT activity which has proven able to replace the MBA in the diagnosis of botulism since its sensitivity is of the same order of magnitude or even better than the MBA for most known toxin serotypes [15,16,21]. EndoPep-MS was initially developed in costly instruments but Perry and coworkers [11] demonstrated that, for the detection of BoNT/A, B, E, and F in clinical samples, it can be is successfully applied to the Bruker MALDI Biotyper, an instrument that has become quite common in microbiology laboratories for bacteria identification. The majority of veterinary botulism cases are caused by *C. botulinum* Group III strains which can produce the closely related toxins BoNT/C and D or their mosaic variants BoNT/CD and BoNT/DC [9,22]. BoNT/CD is composed of a C light chain and a D heavy chain whereas BoNT/DC has a D light chain and a C heavy chain. Therefore, the CD toxin exhibits the typical enzymatic activity of BoNT/C and the DC toxin acts as a BoNT/D [23,24]. To our knowledge, no protocols have previously been developed for the detection of BoNTs of animal interest using the Bruker MALDI Biotyper. We validated and implemented the EndoPep-MS method for diagnosing animal botulism using the Bruker MALDI Biotyper by evaluating analytical and diagnostic sensitivity and specificity using clinical samples and artificially contaminated samples. We also compared the performance of EndoPep-MS based on our protocol with the MBA. The validation process, carried out with BoNT/C and D positive samples and their mosaic variants BoNT/CD and BoNT/DC, demonstrated that the method is able to detect all serotypes with high sensitivity and can replace the MBA in diagnostic practice. Our EndoPep-MS method cannot distinguish between pure sub-types C and D and their mosaic forms. However, although these data are important for clarifying the epidemiology of animal botulism and could have relevance for vaccine strategies, they are irrelevant for diagnostic purposes and are comparable to what is obtained with the MBA. In addition, the BoNT subtypes can be distinguished by coupling PCR with BoNT detection in isolated strains or in enrichment cultures. Moreover, the EndoPep-MS showed no cross-reactivity when applied to broth cultures of closely related *Clostridia* spp. strains and neurotoxins-producing clostridia type A, B, E, and F, indicating a high degree of analytical specificity.

In terms of analytical sensitivity, EndoPep-MS was able to detect BoNT/C and D in aqueous solution with an LoD of less than or equal to 1 mLD_50_/500 µL for BoNT/D and between 1 and 2 mLD_50_/500 µL for BoNT/C. Tests conducted with sera, broth cultures of feces and feces diluted to 1:1 with GPB spiked with two-fold dilutions of reference toxins, showed limited or no interference of the matrix in LoD, with the exception of feces spiked with BoNT/C. In the latter case, the presence of nonspecific signals at all tested dilutions prevented detection of specific peaks related to the cleaved substrates. Superimposable results were also obtained when a different peptide, the Pep 39 [21], was used (data not shown). However, this interference in cleaved peptide detection was not observed in the only fecal sample tested, collected from a swan which died from botulism (sample 5641/12, Table 4). It is well known that in complex matrices, such as feces, the high amount of endogenous proteases may digest peptide substrates before reacting with BoNT, leading to false results. It is commonly reported that the sensitivity of EndoPep-MS is lower in stool extracts than in other clinical samples [15]. Nevertheless, EndoPep-MS proved to be appropriate to detect BoNT/A, B, E, and F in clinical samples since their concentration in stools can reach very high levels [15,25]. The amount of BoNT/C in the swan fecal sample was not quantified but the clear positivity found in the specimen suggests that the BoNT concentration was high enough to overcome the effect of endogenous proteases. One further explanation for the difference between the MBA and the EndoPep-MS results for the artificially contaminated samples and fecal sample analyzed could be that the composition of the feces, and therefore the added presence of other types of inhibitors or proteases, varies among animal species and within the same species as well as with type of nutrition and digestive physiology. In our case, the experimental tests were performed using dog feces while the fecal sample was obtained from a swan. Dog feces contain very high levels of proteases, and therefore we decided to test the protocol using a very challenging matrix.

Assaying specimens collected in suspected outbreaks of human and animal botulism by EndoPep-MS showed that the method possesses a sensitivity of 100% and a specificity of 96.08%. This low specificity seems to be due to two false positive results. However, one sample was a serum collected from a bovine that tested positive for *C. botulinum* type C by MBA and qPCR in rumen. The other was a serum sample obtained from a meat chicken from a flock in which other animal sera had tested positive for BoNT/C on MBA. In both cases, the EndoPep-MS was yielded weak positivity in all replicates suggesting that the method could also be more sensitive than the MBA.

## 4. Conclusions

The tests carried out showed that the EndoPep-MS method, initially developed for use with more powerful mass spectrometers, can be applied to the Bruker MALDI Biotyper instrument, showing excellent results in the detection of biological activity of C and D toxins and their mosaic forms. We have demonstrated that our EndoPep-MS protocol provides rapid results which are comparable with the MBA, considered the “gold standard” for BoNT detection. For this reason, EndoPep-MS is a valid alternative to the MBA and can be easily performed in a clinical microbiology laboratory without advanced skills in mass spectrometry.

## 5. Materials and Methods

### 5.1. Materials and Reagents

BoNT/C and BoNT/D toxin complex were obtained using 1 mg/mL stocks from Metabiologics Inc. (Madison, Wisconsin) at the specific toxicity of 6.5 × 10^6^ for BoNT/C and 3 × 10^7^ mLD_50_/mg for BoNT/D. Pep-C and Pep-D/F were synthesized and purchased from GenScript Biotech (Piscataway, NJ, USA). Peptide sequences are reported in Table 5. Antibodies 8DC1.2 (serotypes C, CD, and DC) and 4C2 (serotypes C and DC) were kindly provided by Dr James D. Marks (University of California, San Francisco, CA, USA). Zeba^TM^ Spin Desalting Columns and sulfo-NHS-biotin were purchased from Thermo Fisher Scientific (Rockford, IL, USA). All reagent for the MALDI Biotyper analysis were acquired from Bruker Daltonics (Bremen, Germany). Magnetic Dynabeads M-280 streptavidin were obtained from Invitrogen-Life technologies (Life Technologies AS, Oslo, Norway). Reagents for preparing the reaction buffer (RB) and HBS-EP Buffer were purchased from Sigma-Aldrich (St. Louis, MO, USA).

### 5.2. Coupling of Antibodies with Magnetic Beads

A buffer exchange from Tris Glicina Buffer to PBS was performed on the antibody using Zeba^TM^ Spin Desalting Columns in accordance with the manufacturer’s instructions. The antibodies were then biotinylated by adding 1.0 μL of 0.533 mM sulfo-NHS-biotin to 8.0 μg of antibody. The reaction was carried out over night at room temperature. Subsequently, 2.3 μL of each biotinylated antibody were incubated with 1 mg of Dynabeads^®^ M-280 Streptavidin suspended in 400 µL of HBS-EP buffer and incubated while shaking for one hour at room temperature. Lastly, conjugated magnetic beads were washed twice with 500 μL HBS-EP Buffer (0.01 M HEPES pH 7.4, 0.15 M NaCl, 3 mM EDTA, 0.005% *v/v* Surfactant P20) and resuspended in HBS-EP Buffer at the same volume in which they had been initially suspended.

### 5.3. Enrichment Culture for Detection of Neurotoxin-Producing CLOSTRIDIA

Enrichment cultures were performed as described in the procedure CNRB 30.010 (2017) issued by the National Reference Center for Botulism [26]. Briefly, 1 g or 1 mL of sample or 1 mL of spores (specificity assay), were introduced into 10 mL of pre-reduced Fortified Cooked Meat Medium (FCMM) that was subsequently heated to 70 °C for 10 min and incubated at 30 °C for 4 days in anaerobic conditions.

### 5.4. Sample Preparation for EndoPep-MS Detection

Two grams of stool were mixed at ratio of 1:1 (stool:buffer) with GPB and vigorously vortexed for 5 min. The sample was then centrifuged at 8000× *g* at 4 °C for 20 min and the supernatant collected for further analysis. Liquid samples (sera, milk, broth culture) were centrifuged and the supernatants collected and processed as described below for the toxin detection.

### 5.5. Sample Preparation to Determine the LoD of the EndoPep-MS Method

The samples used for the contamination assay with reference toxins were water, sera, broth culture of dog feces, and dog feces at a dilution of 1:1 in GPB that had previously been analyzed for suspected botulism, and had tested negative by the multiplex real-time PCR [18] and MBA [26]. All specimens were centrifuged at 8000× *g* at 4 °C for 20 min and the supernatants collected and contaminated with 2 µL/500 µL of serial 2-fold dilutions of BoNT/C and BoNT/D reference toxins from 1024 mLD_50_/µL to 0.0625 mLD_50_/µL. Biosafety level-2 practices, processes, and facilities were used to ensure safety while working with BoNTs. Additionally, toxin stock material and all samples containing BoNTs were processed in a Class II biosafety cabinet containing HEPA filters to minimize the potential for aerosol exposure.

### 5.6. Toxin Concentration and Endo-Peptidase Activity Assay

BoNTs were extracted from 500 μL of sample supernatants, using 20 μL of beads coated with specific antibodies. To prevent beads aggregation during the procedure, 50 μL of PBST 10× (0.1 M PBS, 0.5% Tween^®^20) were added to each sample. Toxin binding was carried out while shaking for 1 h at room temperature in a SB2fixed speed rotator (Stuart ^TM^).

Beads were then captured using a magnetic holder and washed twice with 1 mL of PBST, once with 150 μL of PBST, and lastly with 80 μL of ultra-pure water. The water was then completely removed, the beads were suspended in 18 μL of RB buffer (0.1 M DTT, 0.2 mM ZnCl_2_, 1.0 mg/mL BSA, 10 mM HEPES buffer pH 7.3) and incubated with 2 µL of a specific peptide solution at a concentration of 15 mM (Table 5). The reaction buffer and bead mixture were then incubated for 6 h at 42 °C for BoNT/D and overnight at 37 °C for BoNT/C. One microliter of each sample was used for MALDI-TOF analysis.

### 5.7. MALDI-TOF MS Detection

One microliter of each extract was spotted on a MALDI-Biotyper steel target plate, allowed to dry and, subsequently, overlaid with 1 µL of alpha-cyano-4-hydroxycinnamic acid (HCCA) matrix (5 mg/mL HCCA in 50% acetonitrile, 2.5% TFA, 47.5% water) and left to dry completely again. Mass spectra of each spot were obtained using the MALDI- Biotyper Microflex LT instrument (MALDI Biotyper, Bruker Daltonics Inc., Billerica, MA, USA) and the software FlexControl version 3.3 by scanning from 720 to 5000 *m/z* in MS-positive ion linear mode. The laser frequency was set to 30 Hz and the final spectrum was an average of 1000 laser shots (10 groups of 100 shots collected in a spiral pattern).

### 5.8. Limit of Detection (LoD) of the EndoPep-MS Method

Samples contaminated with reference toxins BoNT/C and BoNT/D at final concentrations ranging from 2048 to 0.125 mLD_50_/500 µL were analyzed in triplicate by the EndoPep-MS method (technical replicates) as previously described. The experiment was repeated three times for each matrix (biological replicates). The LoD for each independent experiment was defined as the lowest level of toxin that could be detected in the three technical replicates. Detection of the toxins consisted of the presence of mass spectrometric peaks with S/N greater than 3 times the S/N of the negative control (unspiked sample of the same matrix).

### 5.9. Specificity of the EndoPep-MS Method

The analytical specificity of the EndoPep-MS method was evaluated by spiking broth cultures of fecal samples that had tested negative for BoNTs by both the MBA and EndoPep-MS, with reference strains of *Clostridium* spp. related to *C. botulinum* or commonly found in animal feces and known to cause disease. Clostridia strains producing BoNT/A, B, E, and F were also included (Table 2). After 48 h of incubation in anaerobic conditions at 30 ± 1 °C the supernatant of all cultures were analyzed by EndoPep-MS, multiplex real-time PCR [18], and MBA [26]. To limit the number of mice used for research purposes, only three mice with untreated and heat-treated culture supernatant were inoculated. No samples treated with antitoxins specific for each BoNT were tested.

### 5.10. Comparison of the LoD of EndoPep-MS and MBA

Broth cultures of feces, sera, and feces previously testing negative for *C. botulinum* neurotoxins were spiked with 8, 4, 2, 1, and 0.5 mLD_50_/500 µL of BoNT/C and BoNT/D, respectively. Samples were then diluted in GPB at ratio of 1:1, reaching a final concentration of 4, 2, 1, 0.5, and 0.25 mLD_50_/500 µL, respectively. Samples were subsequently centrifuged at 8000× *g* at 4 °C for 20 min, the supernatants were collected, split into two portions, and analyzed by both EndoPep-MS in triplicate and by the MBA, following the CNRB 30.011 rev.1 method with some modifications [26]. Briefly, 500 µL of each supernatant were injected intraperitoneally into two *Mus musculus* CD-1 mice of 20–30 g which had been observed for at least four days. Death, with the development of signs of botulism intoxication, was recorded. Data were compared with the EndoPep-MS results. To reduce the number of mice required for the comparison study neither neutralized extract nor negative controls were injected, and the experiment was performed one time only for each matrix.

### 5.11. Evaluation of Diagnostic Sensitivity and Specificity of EndoPep-MS

Seventy-nine samples collected during routine diagnostic laboratory analysis (Table 4) and belonging to suspected cases of botulism were analyzed by the EndoPep-MS method following the aforementioned protocols. Food extracts with GPB, broth culture, sera, milk, and feces were included among the 79 samples. Each sample was analyzed twice and reported to be positive when both replicates were positive on EndoPep-MS. Sensitivity, specificity, positive predictive value, negative predictive value, and accuracy of the EndoPep-MS method were calculated using MedCalc Statistical Software version 19.2.6 (MedCalc Software Ltd., Ostend, Belgium; https://www.medcalc.org; 2020) by means of the Diagnostic test option (2 × 2 table).

### 5.12. Multiplex Real-Time PCR to Detect BoNT/C and D and Their Mosaic Forms.

Evaluation of the BoNT serotypes was carried out by the multiplex real-time PCR method issued by the National Reference Center for Botulism, CNRB31.010 (2017) [18].

### 5.13. Ethic Approval

The MBA for the development of the EndoPep-MS method was authorized by the Italian Ministry of Health (authorization No. 239/2015-PR) on 9 April 2015. The laboratory is also officially authorized by the Italian Ministry of Health to use animals for diagnostic purposes (authorization No. n. 243/2020).

## Figures and Tables

**Table 1:** Limit of detection LoD of EndoPep-MS in samples spiked with BoNT/C and BoNT/D reference toxins.

BoNT	Matrix	Biological Replicates		
		1	2	3
		LoD (mLD_50_/500 µL)	LoD (mLD_50_/500 µL)	LoD (mLD_50_/500 µL)
C	Water	1	2	2
	Sera	2	1	2
	Broth culture	2	2	1
	Feces	N/A	N/A	N/A
D	Water	1	0.5	0.5
	Sera	0.5	0.5	0.25
	Broth culture	2	1	2
	Feces	2	1	2

N/A: not applicable.

**Table 2:** EndoPep-MS specificity studies. Results of EndoPep-MS assay, MBA and qPCR applied to broth cultures inoculated with different *Clostridium* spp. and *C. botulinum* type A, B, E, F, C, CD, DC, and D strains.

Strains	Reference N.	EndoPep-MS		qPCR *	MBA **
		Peptide C	Peptide D		
*C. botulinum* type A	ATCC 19397	-	-	A	Expired
*C. botulinum* type B	CCUG 7969	-	-	B	Expired
*C. butyricum* type E	DSM 10702	-	-	E	Expired
*C. botulinum* type F	NCTC 10281	-	-	F	Expired
*C. botulinum* type C	9877/1/12	+	-	C	Expired
*C. botulinum* type C/D	6503/1/13	+	-	C/D	Expired
*C. botulinum* type D/C	3522/5/13	-	+	D/C	Expired
*C. botulinum* type D	4150/24/18	-	+	D	Expired
*Clostridium sordellii*	ATCC 9714	*-*	*-*	Negative	Survived
*Clostridium tetani*	ATCC 10779	-	-	Negative	Survived
*Clostridium sporogenes*	DSM 795	-	-	Negative	Survived
*Clostridium difficile*	ATCC 9689	-	-	Negative	Survived
*Clostridium haemolyticum*	ATCC 9650	-	-	Negative	Survived
*Clostridium novyi*	ATCC 25758	-	-	Negative	Survived

(+) Detection of peaks related to cleaved peptides; (-) Absence of peaks related to the cleaved peptide. * qPCR: Multiplex real time PCR method [18]; ** Only untreated supernatants and heat-treated ones were tested; Expired: presence of heat-labile neurotoxin, Survived: absence of heat-labile neurotoxin.

**Table 3:** Comparison of the MBA and EndoPep-MS using spiked samples.

Concentration		EndoPep-MS			MBA	
(mLD_50_/500 µL)		Replicate 1	Replicate 2	Replicate 3	Mouse 1	Mouse 2
			**BoNT/C**			
**Serum**	**4**	Positive	Positive	Positive	Expired	Expired
	**2**	Positive	Positive	Positive	Expired	Expired
	**1**	Positive	Positive	Positive	Expired	Expired
	**0.5**	Negative	Negative	Negative	Expired	Expired
	**0.25**	Negative	Negative	Negative	Survived	Survived
	**Negative**	Negative	Negative	Negative	NT	NT
**Broth culture**	**4**	Positive	Positive	Positive	Expired	Expired
	**2**	Positive	Positive	Positive	Expired	Survived
	**1**	Negative	Negative	Negative	Survived	Survived
	**0.5**	Negative	Negative	Negative	Survived	Survived
	**0.25**	Negative	Negative	Negative	Survived	Survived
	**Negative**	Negative	Negative	Negative	NT	NT
**Feces (1:1 PGB)**	**4**	N/A	N/A	N/A	Expired	Expired
	**2**	N/A	N/A	N/A	Survived	Survived
	**1**	N/A	N/A	N/A	Survived	Survived
	**0.5**	N/A	N/A	N/A	Survived	Survived
	**0.25**	N/A	N/A	N/A	Survived	Survived
	**Negative**	N/A	N/A	N/A	NT	NT
**BoNT/D**						
**Serum**	**4**	Positive	Positive	Positive	Expired	Expired
	**2**	Positive	Positive	Positive	Expired	Expired
	**1**	Positive	Positive	Positive	Survived	Survived
	**0.5**	Positive	Positive	Negative	Survived	Survived
	**0.25**	Negative	Negative	Negative	Survived	Survived
	**Negative**	Negative	Negative	Negative	NT	NT
**Broth culture**	**4**	Positive	Positive	Positive	Expired	Expired
	**2**	Positive	Positive	Positive	Expired	Expired
	**1**	Negative	Negative	Negative	Expired	Expired
	**0.5**	Negative	Negative	Negative	Survived	Survived
	**0.25**	Negative	Negative	Negative	Survived	Survived
	**Negative**	Negative	Negative	Negative	NT	NT
**Feces (1:1 PGB)**	**4**	Positive	Positive	Positive	Expired	Expired
	**2**	Positive	Positive	Positive	Expired	Survived
	**1**	Negative	Negative	Negative	Survived	Survived
	**0.5**	Negative	Negative	Negative	Survived	Survived
	**0.25**	Negative	Negative	Negative	Survived	Survived
	**Negative**	Negative	Negative	Negative	NT	NT

N/A: not applicable; NT: not tested.

**Table 4:** Comparison between the MBA, EndoPep-MS method, and qPCR applied in positive and negative diagnostic samples.

Sample ID	Origin	BoNTs		EndoPep-MS	
		MBA	qPCR	BoNT/C	BoNT/D
**GPB extracts**					
8/4/15	Vegetables + meat	NEG	/	NEG	NEG
8/3/15	Vegetables + meat	NEG	/	NEG	NEG
8/6/14	Vegetables + meat	BoNT/E	/	NEG	NEG
8/7/14	Vegetables + meat	BoNT/A	/	NEG	NEG
8/8/15	Vegetables + meat	BoNT/A	/	NEG	NEG
8/10/15	Vegetables + meat	BoNT/A	/	NEG	NEG
8/9/15	Vegetables + meat	NEG	/	NEG	NEG
8/1/15	Vegetables + meat	NEG	/	NEG	NEG
3/1/15	Vegetables + meat	BoNT/B	/	NEG	NEG
8/2/15	Vegetables + meat	BoNT/A	/	NEG	NEG
**Enrichment cultures**					
45/2/12	Mushrooms in oil	NEG	/	NEG	NEG
3/1/15	Eggplants in oil	BoNT/B	B	NEG	NEG
43/1/12	Human feces	NEG	/	NEG	NEG
19/10/11	Rectal swab	BoNT/E	E	NEG	NEG
1/4/15	Mushrooms in oil	BoNT/B	B	NEG	NEG
2/2/15	Human feces	NEG	/	NEG	NEG
8/8/08	Human feces	BoNT/B	B	NEG	NEG
48/4/12	Human feces	NEG	/	NEG	NEG
18/5/12	Olives	BoNT/B	B	NEG	NEG
18/5/12	Sausages	NEG	/	NEG	NEG
21/19/12	Tuna in oil	NEG	/	NEG	NEG
55/6/11	Eggplants in oil	NEG	/	NEG	NEG
153/113	Truffle cream	NEG	/	NEG	NEG
20/3/12	Chili pepper in oil	NEG	/	NEG	NEG
16/3/12	Human feces	BoNT/B	B	NEG	NEG
166/2/13	Human feces	NEG	/	NEG	NEG
1556/15	Tuna	BoNT/B	B	NEG	NEG
16/4/12	Mushrooms in oil	BoNT/B	B	NEG	NEG
22/2/14	Human feces	BoNT/B	B	NEG	NEG
22/4/14	Human feces	BoNT/B	B	NEG	NEG
46/3/14	Human feces	BoNT/B	B	NEG	NEG
152/1/10	Truffle cream	BoNT/B	B	NEG	NEG
47/2/15	Human feces	BoNT/B	B	NEG	NEG
11/3/15	Mushrooms in oil	BoNT/B	B	NEG	NEG
18/2/15	Human feces	BoNT/B	B	NEG	NEG
42/2/15	Human feces	BoNT/B	B	NEG	NEG
4871/15	Human feces	NEG	/	NEG	NEG
49/2/15	Human feces	BoNT/B	B	NEG	NEG
5271715	Chili pepper + tuna in oil	NEG	/	NEG	NEG
5509/1/20	Wood shavings	BoNT/C + BoNT/D	C + D	POS	POS
5509/2/20	Litter	BoNT/C + BoNT/D	C + D	POS	POS
4888/20	Quail	BoNT/D	D/C	NEG	POS
5291/1/20	Water (drinking)	BoNT/D	D/C	NEG	POS
5291/2/20	Water (tank)	BoNT/D	D/C	NEG	POS
5729/1/20	Bovine	BoNT/D	D	NEG	POS
5729/2/20	Bovine	BoNT/D	D	NEG	POS
6126/2/20	Water	BoNT/D	D	NEG	POS
6126/3/20	Water	BoNT/D	D	NEG	POS
6126/7/20	Quail	BoNT/D	D	NEG	POS
6126/8/20	Quail	BoNT/D	D	NEG	POS
6126/10/20	Quail	BoNT/D	D	NEG	POS
6126/11/20	Quail	BoNT/D	D	NEG	POS
**Sera**					
5674/10	Duck	BoNT/C	/	POS	NEG
8103/2/09	Broiler	BoNT/C	/	POS	NEG
8103/10/09	Broiler	NEG	/	NEG	NEG
8103/13/09	Broiler	BoNT/C	/	POS	NEG
2659/2/10	Broiler	NEG	/	NEG	NEG
2659/4/10	Broiler	NEG	/	POSw	NEG
2659/5/10	Broiler	BoNT/C	/	POS	NEG
4691/11	Turkey	BoNT/C	/	POS	NEG
4863/13	Pheasant	BoNT/C	/	POS	NEG
474/14	Dog	BoNT/C	/	POS	NEG
5313/28/14	Broiler	BoNT/C	/	POS	NEG
5313/29/14	Broiler	BoNT/C	/	POS	NEG
5313/30/14	Broiler	BoNT/C	/	POS	NEG
6289/3/14	Mallard	NEG	/	NEG	NEG
6289/4/14	Mallard	NEG	/	NEG	NEG
5993/15	Pheasant	BoNT/C	/	POS	NEG
6353/15	Broiler	BoNT/C	/	POS	NEG
6660/1/15	Broiler	BoNT/C	/	POS	NEG
6660/2/15	Broiler	BoNT/C	/	POS	NEG
6841/2/15	Bovine	NEG	/	NEG	NEG
6841/3/13	Bovine	NEG	/	POS_w_	NEG
**Feces**					
5641/12	Swan	BoNT/C	/	POS	NEG
**Milk**					
9861/51/18	Bovine	NEG	/	NEG	NEG
9861/189/18	Bovine	NEG	/	NEG	NEG
9861/242/18	Bovine	NEG	/	NEG	NEG
9861/251/18	Bovine	NEG	/	NEG	NEG
9861/258/18	Bovine	NEG	/	NEG	NEG

_w_ = weak; POS: positive; NEG: negative.

**Table 5:** Peptide substrates amino acid sequence used for specific BoNT detection and respective mass of uncleaved and cleaved substrates.

Toxin Type	Peptide		*m/z*		Reference
	Name	Sequence			
BoNT/C	PepC	KGSNRTIDEANQRA/ATRMLGGK-biotin	[M + H]^+^	2912.3	[27]
			[M + 2H]^2+^	1457.1	
			CT	1059.5	
			NT	1870.9	
BoNT/D	Pep D/F	TSNRRLQQTQAQVDEVVDIMRVNVDKVLERDQK/LSELDDRADAL	[M + H]^+^	/	[14]
			[M + 2H]^2+^	2605.4	
			CT	1217.3	
			NT	4013.5	

## Data Availability

The raw data generated and analysed during the current study are available from the corresponding author I.D. on reasonable request.
